# Hydrochlorothiazide Test as a Tool in the Diagnosis of Gitelman Syndrome in Chinese Patients

**DOI:** 10.3389/fendo.2018.00559

**Published:** 2018-09-24

**Authors:** Xiaoyan Peng, Bingbin Zhao, Lei Zhang, Lanping Jiang, Tao Yuan, Ying Wang, Haiyun Wang, Jie Ma, Naishi Li, Ke Zheng, Min Nie, Xuemei Li, Xiaoping Xing, Limeng Chen

**Affiliations:** ^1^Department of Nephrology, Peking Union Medical College Hospital, Peking Union Medical College, Chinese Academy of Medical Sciences, Beijing, China; ^2^Department of Endocrinology, Peking Union Medical College Hospital, Peking Union Medical College, Chinese Academy of Medical Sciences, Beijing, China

**Keywords:** Gitelman syndrome, diagnosis, hypomagnesemia, hypocalciuria, hydrochlorothiazide test

## Abstract

Traditional clinical diagnostic criteria for Gitelman syndrome (GS) including hypomagnesemia and hypocalciuria have been challenged by reports of atypical manifestations recently, as well as the development of genetic testing. Hydrochlorothiazide (HCT) test is a diagnostic method different from the traditional biochemical parameters, which could evaluate the function of thiazide-sensitive sodium-chloride co-transporter (NCC) *in vivo* by a small dose of NCC inhibitor HCT. In this retrospective study, we compared the diagnostic significance of hypomagnesemia, hypocalciuria, and the reaction of HCT test, among Chinese patients with GS confirmed by genetic test. For patients who were clinically suspected of GS manifestations, *SLC12A3* gene was sequenced to make genetic diagnosis. A total of 83 GS and 19 control patients were recruited, among which 37 underwent HCT test according to the standard process. Compared with the gold standard of genetic diagnosis, both the diagnostic sensitivity (93.10%) and specificity (100.00%) of the HCT test were much higher than those of hypomagnesemia and/or hypocalciuria. The area under the receiver operating characteristic (ROC) curve was 1.000 (95% CI 0.905–1.000) for HCT test, higher than the values using hypomagnesemia and/or hypocalciuria. The cost of HCT test was around $54, much lower than genetic diagnosis. In conclusion, besides traditional hypomagnesemia and hypocalciuria, HCT test could be a valuable tool in the clinical diagnosis of Chinese GS patients.

## Introduction

Gitelman syndrome (GS) (OMIM 263800) is a recessively inherited salt-losing renal tubulopathy. It is caused by loss-of-function mutations in *SLC12A3* which encodes the thiazide-sensitive sodium-chloride co-transporter (NCC) located in the apical membranes of the distal convoluted tubule (DCT) cells (1). The disease is characterized by hypokalemic metabolic alkalosis, usually accompanied by hypomagnesemia and hypocalciuria (2). Prior studies reported that GS patients showed a high phenotypic variability from mild weakness or nausea to life-threatening complications, such as ventricular arrhythmia. Most of them complained of poor quality of life (3). Therefore, it is challenging yet critical to establish a reliable method for diagnosing GS.

Current gold standard for diagnosing GS is genetic testing. It was reported to have a 100% specificity and a sensitivity of 90–100% (1). However, given its high cost and technical requirements, it is difficult to advocate genetic testing in community-level hospitals, especially in resource-limited areas. Moreover, exon sequencing may fail to detect mutations in introns or gene regulatory areas, thus limiting its sensitivity (4, 5). Therefore, the clinical diagnostic strategy of GS is still the cornerstone of the patient care.

Since 1966, hypomagnesemia and hypocalciuria are classic biochemical derangements and the most commonly used criteria to differentiate GS from Bartter syndrome (BS) (6). However, a few GS cases with normomagnesemia or normocalciuria have been reported recently (7–9). In our previous study, GS patients with normomagnesemia demonstrated milder clinical manifestations in comparison with those with hypomagnesemia (10, 11). Hydrochlorothiazide (HCT) test is another clinical diagnostic method that is distinguished from traditional biochemical parameters by its ability to directly evaluate the function of NCC *in vivo* using moderate-dose HCT, an inhibitor of NCC (12). In Chinese GS patients, we have established the cutoff value for chloride fractional excretion in HCT test modified from established methods by Colussi et al. (11–13). However, because of the limited sample size, the predictive value of the currently available diagnostic criteria for GS remains uncertain. According to our knowledge, no study has systematically assessed the accuracy of clinical diagnostic strategies in a large cohort of GS patients. Thus, the aim of the present study is to evaluate the diagnostic significance of hypomagnesemia, hypocalciuria and HCT test, in Chinese GS patients.

## Materials and methods

### Patient recruitment and clinical evaluation

**Figure 1:**
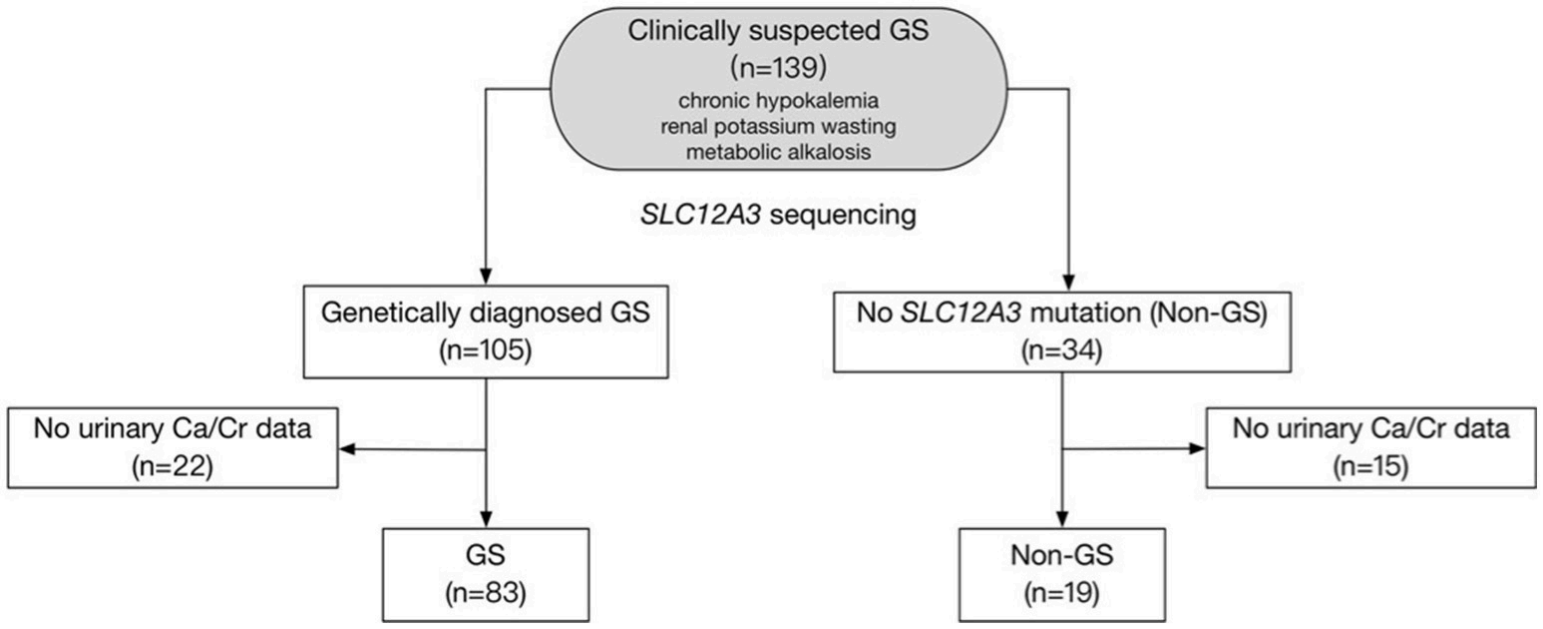
Study flow diagram. The diagram shows how patients were included and excluded from the target population. A total of 102 patients were finally enrolled in this study.

This is a retrospective study. A total of 139 clinically suspected GS patients with chronic hypokalemia, renal potassium wasting, and metabolic alkalosis were evaluated between 2005 and 2017 in Peking Union Medical College Hospital, Beijing, China (Figure 1). Inclusion required presence of the following criteria: (1) Chronic hypokalemia (<3.5 mmol/L) with inappropriate renal potassium wasting (24 h urinary potassium-to-creatinine ratio >2.0 mmol/mmol); (2) Metabolic alkalosis; (3) Low or normal-low blood pressure (<140/90 mmHg). Exclusion criteria included deficiency of potassium intake, surreptitious vomiting, chronic use of diuretics or laxatives, other causes of volume depletion, and secondary causes of tubular dysfunction. The authors adhered to the Declaration of Helsinki, and patients were enrolled after providing informed consent.

### *SLC12A3* gene sequencing

For all 139 patients, the *SLC12A3* gene sequencing was performed as previously described for genetic testing (11). Briefly, the genomic DNA isolated and purified from peripheral blood lymphocytes was used for polymerase chain reaction with a total of 23 pairs of oligonucleotide primers to amplify all 26 exons and flanking intronic regions of *SLC12A3* gene. Sanger direct sequencing was performed on an ABI3730xl automated DNA sequencer (Life Technologies Division, Applied Biosystems, Foster City, CA) by BGI (Beijing, China). The GenBank sequence with the accession number NM_000339.2 was used as a reference sequence.

### Clinical and biochemical characteristics

Serum and 24-h urinary electrolytes and creatinine were measured during the initial clinic visit. The minimum serum potassium (K), sodium (Na), and chloride (Cl) levels were collected from the medical record. Patients with no urinary calcium-creatinine ratio data were excluded (*n* = 37). We calculated the estimated glomerular filtration rate (eGFR) using the Chronic Kidney Disease-Epidemiology Collaboration equation (CKD-EPI) (14). Hypomagnesemia was defined as any recorded serum magnesium level below 0.7 mmol/L. Hypocalciuria was defined as any recorded molar urinary calcium-to-creatinine ratio from 24-h urine collection below 0.2 mmol/mmol (1).

### HCT test

HCT test was performed according to the standard protocol as previously described (11, 13). Patients were asked to stop taking spironolactone at least 7 days and potassium and magnesium supplementation 1 day prior to the test. After overnight fasting, the patients were asked to remain supine and drink 10 mL water per kilogram of body weight 15 min prior to the test, followed by 150 mL per hour until the end of the test. After two 30-min basal clearances, hydrochlorothiazide (HCT, 50 mg orally) was administered and six additional 30-min clearances were performed. Blood samples were collected after 60 and 240 min, and urine was collected every 30 min, which were used for measurement of electrolytes and creatinine. Chloride excretion was quantified using fractional excretion (FE, with creatinine as a GFR marker) using the formula: FE_Cl_ = (U_Cl_/S_Cl_) × (S_Cr_/U_Cr_) × 100%, where S_Cr_ and U_Cr_ represent serum and urinary creatinine, respectively. We defined blunt HCT test for GS as the net increase in chloride fractional excretion (ΔFE_Cl_) below 2.86%. Among 102 eligible individuals, 29 GS patients and 8 non-GS patients received HCT test. The majority of patients declined the test as genetic sequencing is free and convenient in our hospital, and they have received genetic test and considered HCT test to be unnecessary. We also conducted a preliminary cost analysis for HCT test considering disposables used.

### Statistics

Data were expressed as mean ± SD, median (25, 75th) or percentages. The data fitting normal distribution were assessed using *t*-test. The indicators from 24 h urine were compared using log-rank test. Sensitivity, specificity, positive predictive value [PV(+)], negative predictive value [PV(–)], and area under the receiver operating characteristic (ROC) curves were calculated. The kappa coefficients were used to test for agreement between these criteria and genetic testing-based diagnosis. For the best combination of tests to detect GS in this population, binary multivariate logistic regression through a backward model was established. Differences were considered significant at *P* < 0.05. All statistical analyses were performed with SPSS 17.0 statistical software (SPSS, Chicago, IL).

## Results

### Characteristics of clinical and laboratory data

Between 2005 and 2017, 139 patients received genetic testing in Peking Union Medical College Hospital and 102 were enrolled in this study (Figure 1). Baseline demographic, clinical and biochemical data of the 83 GS and 19 non-GS patients were presented in Tables 1, 2.

**Table 1:** General information and clinical manifestations of genetically confirmed GS and non-GS patients.

**Items**	**GS (*n* = 83)**	**Non-GS (*n* = 19)**	***P-*value**
**GENERAL INFORMATION**			
Male	48 (57.8%)	9 (47.4%)	0.407
Age, years	31.1 ± 13.0	32.0 ± 12.4	0.772
Onset age, years	24.4 ± 13.8	25.2 ± 16.7	0.833
Duration, months	44.0 (6.0, 102.0)	48.0 (8.5, 102.0)	0.626
BMI, kg/m^2^	22.45 ± 3.98	23.29 ± 5.86	0.496
eGFR, mL/min/1.73m^2^	116.5 ± 21.6	101.3 ± 24.0	0.008
SBP, mmHg	109.9 ± 12.3	112.5 ± 18.5	0.482
DBP, mmHg	72.1 ± 9.9	70.6 ± 11.1	0.582
SYMPTOMS^*^^#^			
Muscle weakness	59 (72.0%)	12 (70.6%)	1.000
Fatigue	51 (62.2%)	12 (70.6%)	0.513
Palpitations	45 (54.9%)	8 (47.1%)	0.556
Nocturia	35 (42.7%)	5 (29.4%)	0.310
Paresthesia	34 (41.5%)	3 (17.6%)	0.065
Muscle stiffness/pain	32 (39.0%)	2 (11.8%)	0.031
Carpopedal spasm/tetany	27 (32.9%)	6 (35.3%)	0.851
Thirst	25 (30.5%)	3 (17.6%)	0.439
Polyuria	22 (26.8%)	2 (11.8%)	0.767
Dizziness	20 (24.4%)	6 (35.3%)	0.531
Cramps	17 (20.7%)	5 (29.4%)	0.643
Abdominal pain	9 (11.0%)	0	0.332
Diarrhea	7 (8.5%)	4 (23.5%)	0.172
Fainting	4 (4.9%)	1 (5.9%)	1.000
Arthralgia	4 (4.9%)	2 (11.8%)	0.600

*Values are mean ± SD, median (25th, 75th) or n (%)*.

* *n (GS) = 82, the patient who had suffered drug-induced deafness and was unable to express himself well was excluded from symptom evaluation*.

# *n (Non-GS) = 17, symptoms of 2 patients were absent. BMI, body mass index; eGFR, estimated glomerular filtration rate; SBP, systolic blood pressure; DBP, diastolic blood pressure*.

**Table 2:** Laboratory biochemical data of genetically confirmed GS and non-GS patients.

**Items**	**GS (*n* = 83)**	**Non-GS (*n* = 19)**	***P-*value**	**Reference values**
minimum serum K^*^, (mmol/L)	2.21 ± 0.43	2.55 ± 0.69	0.006	3.5–5.5
minimum serum Mg^*^, (mmol/L)	0.59 ± 0.15	0.82 ± 0.17	< 0.001	0.70–1.10
minimum serum Cl^*^, (mmol/L)	93.1 ± 4.3	95.4 ± 6.0	0.066	96–111
**Serum**^#^**(mmol/L)**				
K	3.15 ± 0.48	3.26 ± 0.57	0.401	3.5–5.5
Na	138.0 ± 3.2	139.1 ± 2.3	0.175	135–145
Cl	96.1 ± 3.7	100.0 ± 4.8	< 0.001	96–111
Mg	0.64 ± 0.16	0.86 ± 0.19	< 0.001	0.70–1.10
Ca	2.41 ± 0.14	2.35 ± 0.17	0.093	2.13–2.70
P	1.25 ± 0.22	1.25 ± 0.22	0.921	0.81–1.45
Creatinine (μmol/L)	67.9 ± 18.1	78.1 ± 15.1	0.025	59–104
24 h Urine(mmol/day)				
K	94.5 (66.0, 121.5)	70.0 (36.4, 103.2)	0.020	
Na	218.4 (166.0, 285.6)	163.0 (101.0, 223.0)	0.005	
Cl	244.8 (188.0, 325.0)	182.0 (116.3, 230.0)	0.005	
Mg	4.46 (3.75, 5.85)	4.28 (3.13, 6.19)	0.723	
Ca	0.98 (0.49, 1.94)	3.33 (2.28, 4.83)	< 0.001	
P	16.11 (10.58, 22.42)	19.23 (12.18, 22.68)	0.529	
Ca/Cr(mmol/mmol)	0.148 (0.047, 0.310)	0.353 (0.166, 0.585)	0.003	0.20–0.57
**Arterial blood gas**				
pH	7.468 ± 0.030	7.428 ± 0.038	< 0.001	7.350–7.450
cHCO${}_{3}^{-}$(mmol/L)	29.56 ± 3.85	26.71 ± 4.28	0.006	22.0–27.0
ABE(mmol/L)	5.45 ± 3.14	2.53 ± 3.99	0.001	−3.0 – +3.0
**Renin-Angiotensin system**				
Renin(ng/mL/h)	2.94 (1.40, 12.00)	2.78 (0.56, 11.00)	0.294	0.93–6.56
AngII(pg/mL)	229.1 (147.1, 386.5)	365.0 (106.9, 633.0)	0.602	25.3–145.3
Ald(ng/dL)	19.61 (13.43, 24.41)	23.54 (15.26, 26.95)	0.356	6.5–29.6
QTc(ms)	443.3 (420.8, 466.3)	404.0 (377.0, 431.0)	0.001	<450

*Values are mean ± SD, median (25th, 75th) or n (%)*.

* *The minimum serum potassium (K), sodium (Na) and chloride (Cl) levels are the minimal levels in the record. #The serum electrolytes levels were measured when the patients visited our hospital for the first time*.

*Ca/Cr urinary calcium-to-creatinine ratio. ABE, actual base excess; AngII, angiotensin II; Ald, aldosterone; QTc, corrected QT interval*.

The average age of GS patients was (31.1 ± 13.0) years and 57.8% were men. Both GS patients and non-GS patients had a wide range of clinical manifestations associated with hypokalemia. The most common symptoms were muscle weakness (72.0 and 70.6%) and fatigue (62.2 and 70.6%). The percentage of muscle stiffness or pain was much higher in GS patients. The serum creatinine and eGFR were both normal in GS patients and non-GS patients. GS patients presented with more prominent hypokalemia and hypomagnesemia, as well as higher urinary excretion of potassium, natrium, chloride and calcium. Metabolic alkalosis and prolonged QTc were also much more severe in GS patients.

**Figure 2:**
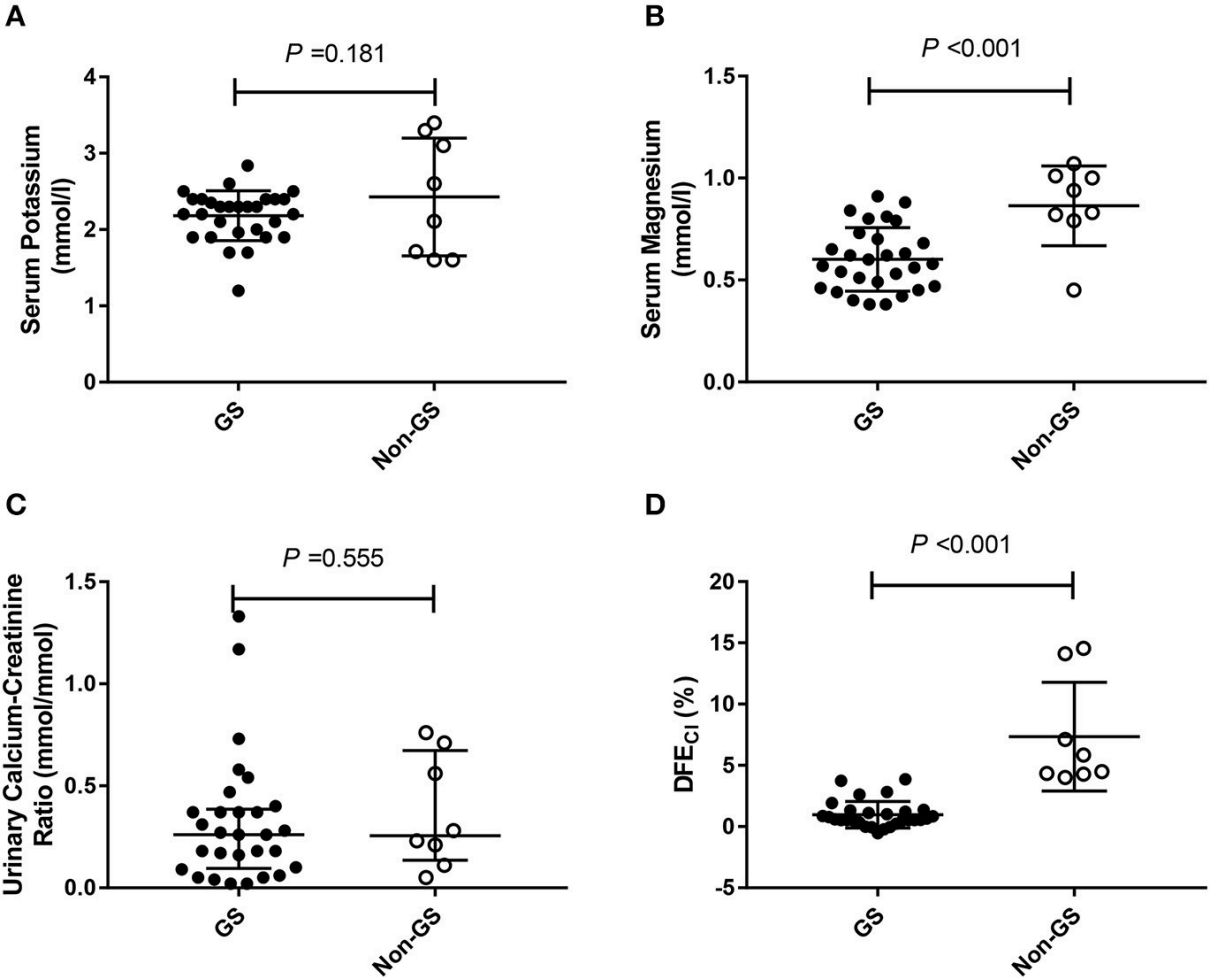
The minimum serum potassium level, the minimum magnesium level, urinary calcium-creatinine ratio and the net increase in chloride fractional excretion (ΔFE_Cl_) in 29 Gitelman syndrome (GS) patients and 8 control patients (non-GS) undergoing HCT test. **(A)** The minimum serum potassium concentrations. **(B)** The minimum serum magnesium concentrations. **(C)** Urinary calcium-creatinine ratio. **(D)** Net increase in chloride fractional excretion (ΔFE_Cl_) in HCT test. HCT test was conducted in 29/83 GS patients and 8/19 non-GS patients. The continuous variables were compared by *t*-test. Abbreviations: GS, Gitelman syndrome; non-GS, patients without GS; ΔFE_Cl_, the net increase in chloride fractional excretion; HCT, hydrochlorothiazide.

Considering that only 37 of the patients underwent HCT test, we compared baseline analysis of patients who underwent HCT test with those who declined the test and found nearly comparable characteristics (Supplemental Tables 1–4). Among patients undergoing HCT tests (***n*** = 37), both GS (***n*** = 29), and non-GS patients (***n*** = 8) had low minimum serum potassium concentration [(2.18 ± 0.33) vs. (2.43 ± 0.77) mmol/L; *P* = 0.181], and low urinary calcium-creatinine ratio [0.264 (0.098, 0.386) vs. 0.253 (0.137, 0.668) mmol/mmol; *P* = 0.555], but GS patients had a significantly lower minimum serum magnesium concentration [(0.60 ± 0.16) vs. (0.86 ± 0.20) mmol/L; *P* < 0.001] and significantly lower ΔFE_Cl_ [(0.97 ± 1.08) vs. (7.34 ± 4.43) %; *P* < 0.001] than non-GS patients (Figure 2).

### Test performances of serum magnesium, urinary calcium-to-creatinine ratio, and HCT test

**Figure 3:**
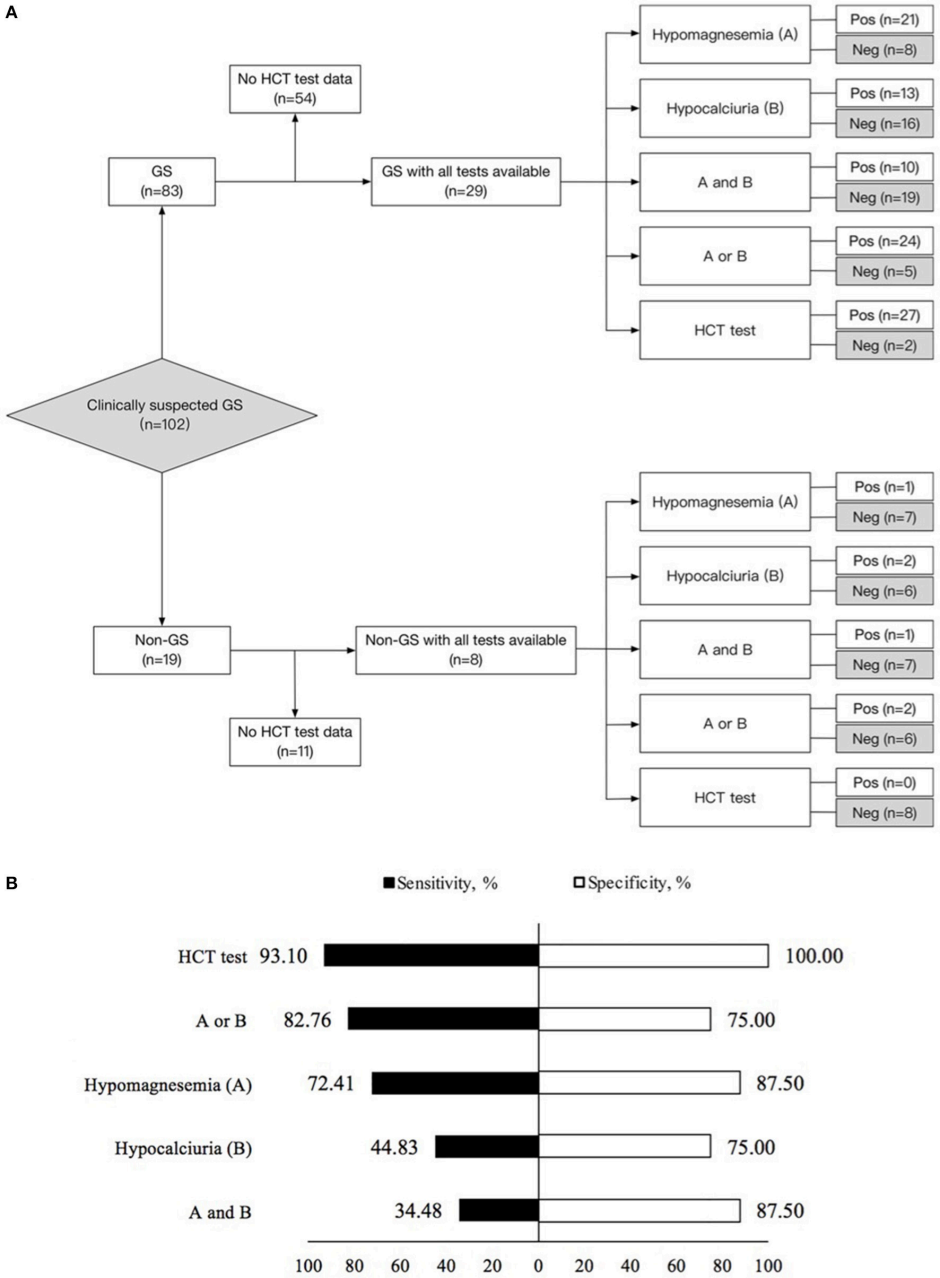
Diagnostic test in 29 Gitelman syndrome (GS) patients and 8 control patients (non-GS) with all tests available. **(A)** Flow diagram of diagnostic test design. **(B)** Sensitivity and specificity for the diagnosis of Gitelman syndrome by means of the five criteria studied. (1) Hypomagnesemia (A); (2) Hypocalciuria (B); (3) Hypomagnesemia and hypocalciuria (A and B); (4) Hypomagnesemia or hypocalciuria (A or B); (5) HCT test. The sensitivity and specificity for each criterion were calculated from the classic 2 × 2 table for comparing a surrogate test to true diagnosis. GS, Gitelman syndrome; urinary Ca/Cr, urinary calcium-creatinine ratio; HCT, hydrochlorothiazide; Pos, positive; Neg, negative.

Using gene sequencing as the gold standard, the sensitivity, specificity, PV (+), PV (–) and kappa value for the diagnosis of GS were analyzed among various tests, including hypomagnesemia, hypocalciuria, hypomagnesemia and hypocalciuria, hypomagnesemia or hypocalciuria, and HCT test. The diagnostic test was first performed in 37 patients with all tests available (Figure 3). HCT test showed the best diagnostic value with a sensitivity of 93.10%, a specificity of 100.00%, and a kappa value of 0.854 (Table 3). Furthermore, we analyzed the accuracy of different diagnosis criteria in 102 patients enrolled, which yielded similar results (Supplemental Figure 1 and Supplemental Table 5). We also conducted a sensitivity analysis excluding the 14 patients previously published in 2015 and HCT test was still the best (Supplemental Table 6).

**Table 3:** Test performances for the diagnosis of Gitelman syndrome by means of the five criteria studied in 37 patients with all tests available.

**Diagnostic performance**	**Hypomagnesemia (A)**	**Hypocalciuria (B)**	**A and B**	**A or B**	**HCT test**
Sensitivity, %	72.41	44.83	34.48	82.76	93.10
Specificity, %	87.50	75.00	87.50	75.00	100.00
PV(+), %	95.45	86.67	90.91	92.31	100.00
PV(-), %	46.67	27.27	26.92	54.55	80.00
Kappa value	0.455	0.121	0.121	0.509	0.854

*PV(+), positive predictive value; PV(-), negative predictive value; HCT, hydrochlorothiazide*.

**Figure 4:**
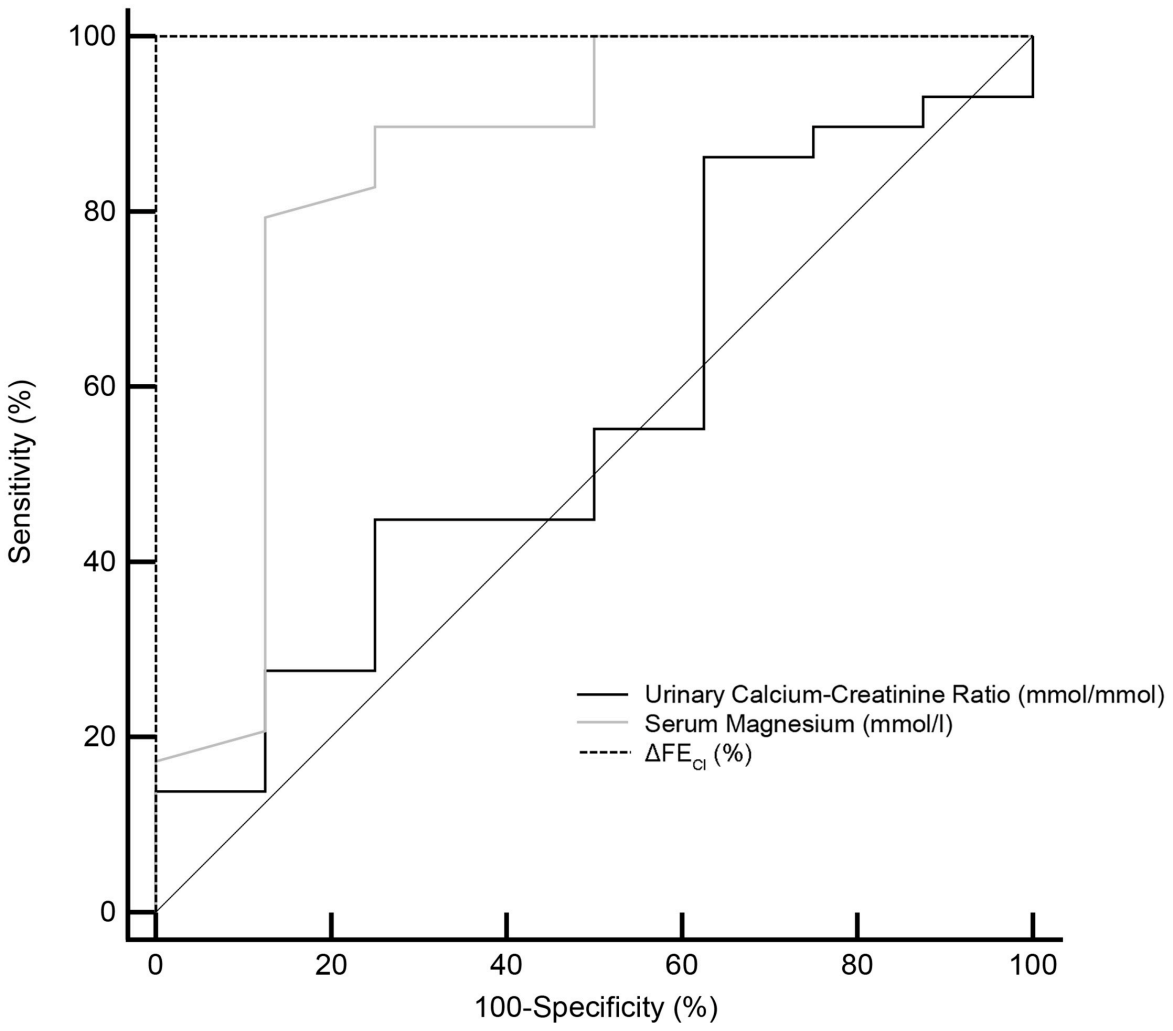
Receiver operating characteristic (ROC) curves for the minimum serum magnesium level, urinary calcium-creatinine ratio and the reaction of hydrochlorothiazide test (ΔFE_Cl_) in the diagnosis of Gitelman syndrome. ROC curve analysis and corresponding area under the curve (AUC) statistics were applied to determine the diagnostic performance. Dotted line for ΔFE_Cl_, gray line for serum magnesium and solid line for urinary calcium-creatinine ratio. AUC, area under the curve; 95% CI, 95% confidence interval; ΔFE_Cl_, the net increase in chloride fractional excretion.

The ROC curves evaluating test performances of serum magnesium, urinary calcium-creatinine ratio and HCT test were shown in Figure 4. The area under the ROC curves (AUC) for HCT test was 1.000 (95% CI 0.905–1.000), higher than that of serum magnesium (0.849, 95% CI 0.693–0.945). Urinary calcium-to-creatinine ratio was not efficient in GS diagnosis with an AUC of 0.569 (95% CI 0.396–0.730). In a larger sample size of 102 patients, the area under the ROC curves (AUC) for magnesium and urinary calcium-to-creatinine ratio was 0.868 (95% CI 0.786–0.927) and 0.719 (95% CI 0.621–0.804), respectively.

**Figure 5:**
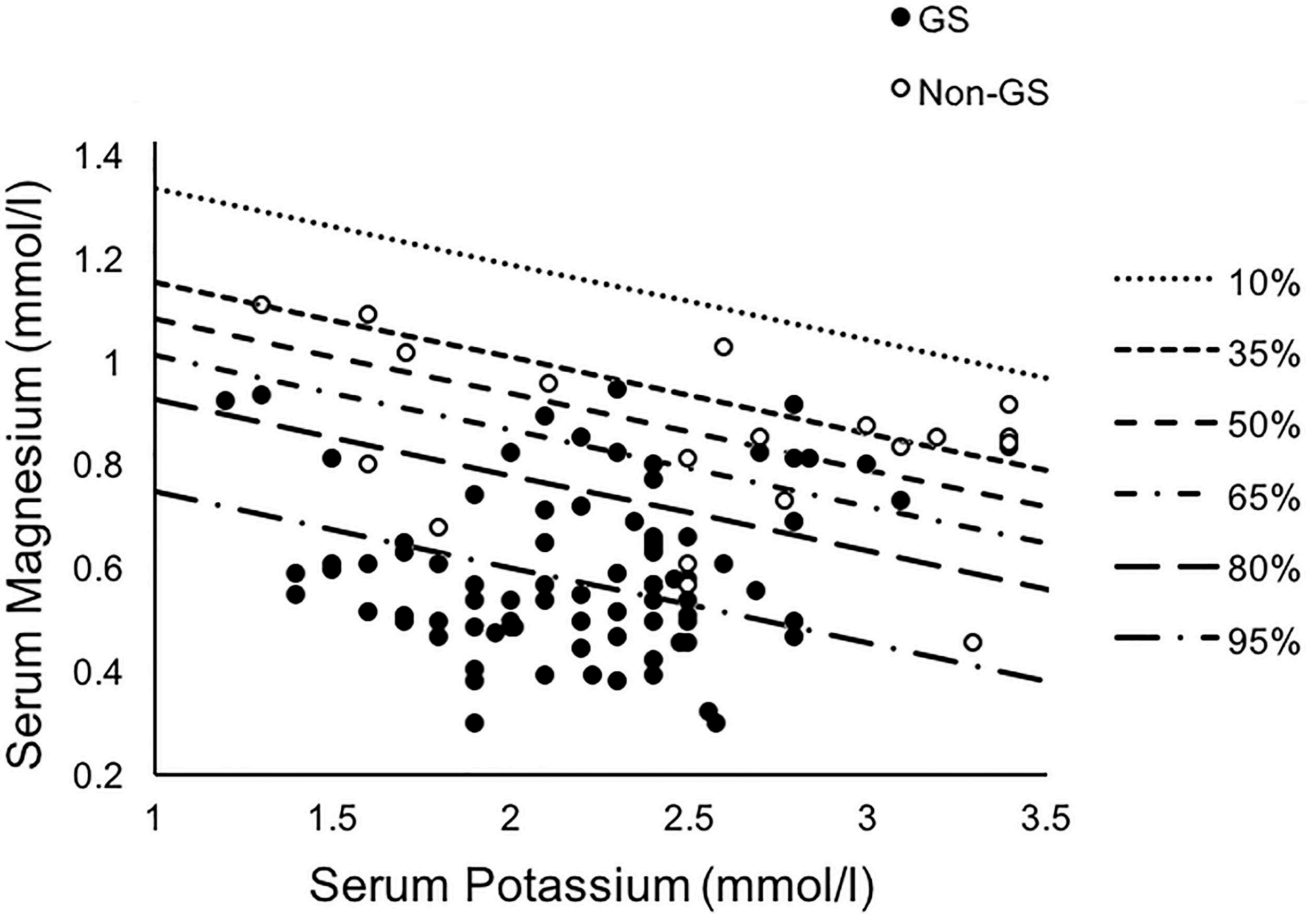
Contour plots displaying the estimated probabilities of diagnosis of Gitelman syndrome in our cohort for the combination model of the minimum serum potassium and magnesium levels. Binary multivariate logistic regression through a backward model was used. GS, Gitelman syndrome; non-GS, patients without GS.

For the combination diagnosis model of the minimum serum potassium and magnesium levels, contour plots were used to display the estimated probabilities of diagnosis of Gitelman syndrome (Figure 5), based on the following probability calculation (15, 16):

$$\begin{array}{l} P=1/\left(1+e^{-10.776+8.794\times \left[Mg\right]+1.283\left[K\right]}\right) \end{array}$$

Among different biochemical markers including serum potassium, magnesium and urinary calcium-to-creatinine ratio, the best combination to achieve the highest combined sensitivity (95.18%), specificity (63.16%), and accuracy (AUC 0.883, 95% CI 0.807–0.960) for GS diagnosis was using minimum serum potassium and magnesium. While the combined model provided the relatively better specificity and sensitivity, the thiazide test was still superior (AUC 1.000, 95% CI 0.905–1.000).

The cost for the HCT test was ¥370 ($54 or €47) per patient (Supplemental Table 7), while genetic testing (direct sequencing, SinoPath, Beijing, China) was 10 times more expensive than HCT test.

## Discussion

In this study, we first demonstrated that the diagnostic efficiency and accuracy of HCT test in Chinese GS patients was much better than hypomagnesemia and/or hypocalciuria.

GS patients are usually characterized by low urinary calcium excretion and hypomagnesemia. But few studies compared the frequency and efficiency of hypomagnemia and hypocalciuria in GS patients. In this GS cohort study, we first concluded that the sensitivity, specificity, and AUC for hypomagnesemia in GS diagnosis were a little better than hypocalciuria. It is different from the previous report from Bettinelli, that GS can be easily distinguished from BS based on the molar ratio of calcium to creatinine rather than plasma magnesium concentration (6). It might be explained by the different genetic mutations associated with racial difference and different cutoff values of hypocalciuria. In Japanese GS patients the ratio of hypocalciuria (81.8%) was higher than our patients, although their cutoff value of hypocalciuria (0.04 mg/mg) was lower than that (0.07 mg/mg) of our patients. Their patients were about 10 years younger than our patients at the age of diagnosis GS (8, 17). On the other hand, urinary calcium excretion was easily affected by increased reabsorption of calcium in both proximal and distal tubules resulted from any course of hypovolemia and the decreased peripheral sensitivity of PTH (18–21).

Hypomagnesemia was also not sensitive enough to diagnose GS. We previously reported the co-localization of NCC and transient receptor potential cation channel subfamily M member 6 (TRPM6) in the kidney (10, 11), and the down-regulation of magnesium channel TRPM6 in GS may explain hypomagnesemia (22). That indicated hypomagnesemia in a certain extent may be secondary to NCC dysfunction. In some cases of GS, hypomagnesemia was not presented at the early stage but appeared years later (23). It is known that circulating magnesium was only 1% of the total body, renal wasting of magnesium would be compensated by releasing of bone and muscle storage. Although the two traditional clinical parameters were not sensitive enough to diagnose GS, if combining serum potassium with magnesium, the diagnostic sensitivity can be improved significantly. The finding may be supported by the correlation between hypomagnesemia and hypokalemia on the basis of both pathophysiologic mechanisms and phenotypic severity in GS patients (10, 24). The intensive mechanism study between the phenotype and genotype would help to reveal truth in the future.

In this study, we proved that HCT test had a higher sensitivity and specificity than serum electrolytes levels and benefited more definite diagnosis of GS as reported before (8, 12). HCT test can be easily conducted with simple protocols and results are available for analysis in a short period of time. Besides, the cost is comparatively low. All the advantages above make it have the potential to be widely used in community-level hospitals. In clinical practice, when patients showed significantly decreased reaction to HCT (i.e., ΔFE_Cl_ < 2.0%), a diagnosis of NCC dysfunction can be made with great confidence, which serves to support subsequent therapy when clinical suspicion is also high. When the result of HCT test is close to the diagnostic threshold, we agree that the diagnosis may be inconclusive and a further furosemide test or genetic test are justified. Our result implicated that screening with HCT test prior to perusing the genetic test (which is both expensive and with limited sensitivity) can be an accurate, cost-effective, and convenient approach to make clinical diagnosis, especially in community-level hospitals or resource-limited areas. Previous studies have reported some practical limitations including poor patient compliance, safety concerns, and potential contraindications to HCT. Factors such as drug interactions, volume status, and dietary electrolyte intake can also affect the test results (1, 12). In the consensus and guideline from KDIGO controversies conference (1), HCT test was not recommended in GS due to its potential related risk such as acute volume depletion and hypersensitivity reactions. In our practice, HCT test was conducted one day after adequate potassium supplement and the patient was closely monitored during the test. During the test, patients' vital signs were stable and no adverse events including symptoms related to hypotension and hypokalemia were observed.

The two GS patients with normal response in HCT test (3.74% and 3.86%) were both male patients with normomagnesemia. One patient carried compound heterozygous mutations with a relatively high baseline fractional excretion of chloride, and his percentage of increase was less than normal. The other patient was heterozygous and had asymptomatic hypokalemia. Variability in diuretic response may be due to underlying mutations and physiologic modulation (12). So, genetic test is still the final confirmatory diagnostic tool (1). For the 19 patients without *SLC12A3* mutation, some underwent further genetic test or clinical inspection, and final diagnosis of Bartter syndrome (BS), hypokalemic periodic paralysis and primary aldosteronism were given. One was found to be an amyloid nephropathy after renal biopsy. A few others remained hypokalemia with unknown cause.

Genetic testing was recommended for all clinically suspected GS patients in KDIGO guideline, but lack of hot-spot mutations, large size of the gene, interpretive challenges of data and limited cost-effectiveness impeded its practical application (1, 25). Previous studies have shown inconsistent results of correlations between phenotype and genotype in GS patients (8, 26, 27). Since HCT test can effectively evaluate NCC function regardless of the specific mutation in *SLC12A3* gene, it may be helpful to connect the genotype with the salt-losing phenotype and narrow the screening population (4, 5). In addition, in patients with GS-like manifestations, a negative HCT test result may justify the use of whole exome sequencing rather than traditional GS and BS gene testing to help identify novel mutation and disease effectively (28).

Our study has several limitations. Firstly, as a retrospective study, the sample size for HCT test is relatively small, which may limit the generalizability of our findings. We have focused on GS gene sequencing since 2005 but first established the HCT test in 2012. It is difficult to call the genetically diagnosed GS patients from all over the country back to our hospital for the HCT test. A few patients declined the HCT test because it is not necessary for their diagnosis when genetic sequence is free and convenient in our hospital. Secondly, the cross-sectional design of our study limited the ability to evaluate the performance of traditional biochemical criteria and function test as screening tools and prognostic factors. A well-designed study, which complies with STARD statement on reporting studies of diagnostic accuracy, may provide more convincing evidence for the advocating of HCT test. We have launched a multi-center prospective GS cohort study recently and we hope to collect more data in the future.

## Conclusion

Hypomagnesemia and hypocalciuria were not accurate enough to diagnose GS in suspected salt-losing tubulopathy. Considering the simple protocol, low cost and few contraindications, HCT test is a valuable tool in the clinical diagnosis of Chinese GS patients, especially in community hospitals of China.

## Author contributions

XP and BZ contribute equally to this manuscript; XP, BZ, LJ, and LC conceptualization; XP and BZ data duration; LC funding acquisition and project administration; XP, LJ, LZ, TY, XX, and LC methodology; XP, BZ, LZ, LJ, TY, YW, HW, JM, NL, KZ, MN, XL, XX, and LC investigation and resources; XP and BZ data analysis; BZ manuscript draft; XP, LZ, and LC manuscript review and editing; LC supervision.

### Conflict of interest statement

The authors declare that the research was conducted in the absence of any commercial or financial relationships that could be construed as a potential conflict of interest.
