# Supplementary material for: Hydrochlorothiazide Test as a Tool in the Diagnosis of Gitelman Syndrome in Chinese Patients
Source: Front Endocrinol (Lausanne). 2018 Sep 24;9:559. doi: 10.3389/fendo.2018.00559 (PMC6165878; doi:10.3389/fendo.2018.00559)
Supplement: Supplementary file 1 [file Data_Sheet_1.doc]

***Supplementary Material***

**Hydrochlorothiazide Test as a Tool in the Diagnosis of Gitelman Syndrome in Chinese Patients**

Xiaoyan Peng, Bingbin Zhao, Lei Zhang, Lanping Jiang, Tao Yuan, Ying Wang, Haiyun Wang, Jie Ma, Naishi Li, Ke Zheng, Min Nie, Xuemei Li, Xiaoping Xing, Limeng Chen*

***Correspondence:** Limeng Chen: chenlimeng@pumch.cn

**Supplemental Figure 1.** Diagnostic test in 102 patients.

(A), Flow diagram of diagnostic test design. (B), Sensitivity and specificity for the diagnosis of Gitelman syndrome by means of the five criteria studied. (1) Hypomagnesemia (A); (2) Hypocalciuria (B); (3) Hypomagnesemia and hypocalciuria (A and B); (4) Hypomagnesemia or hypocalciuria (A or B); (5) HCT test. The sensitivity and specificity for each criterion were calculated from the classic 2×2 table for comparing a surrogate test to true diagnosis. Abbreviations: GS, Gitelman syndrome; HCT, hydrochlorothiazide; Pos, positive; Neg, negative.


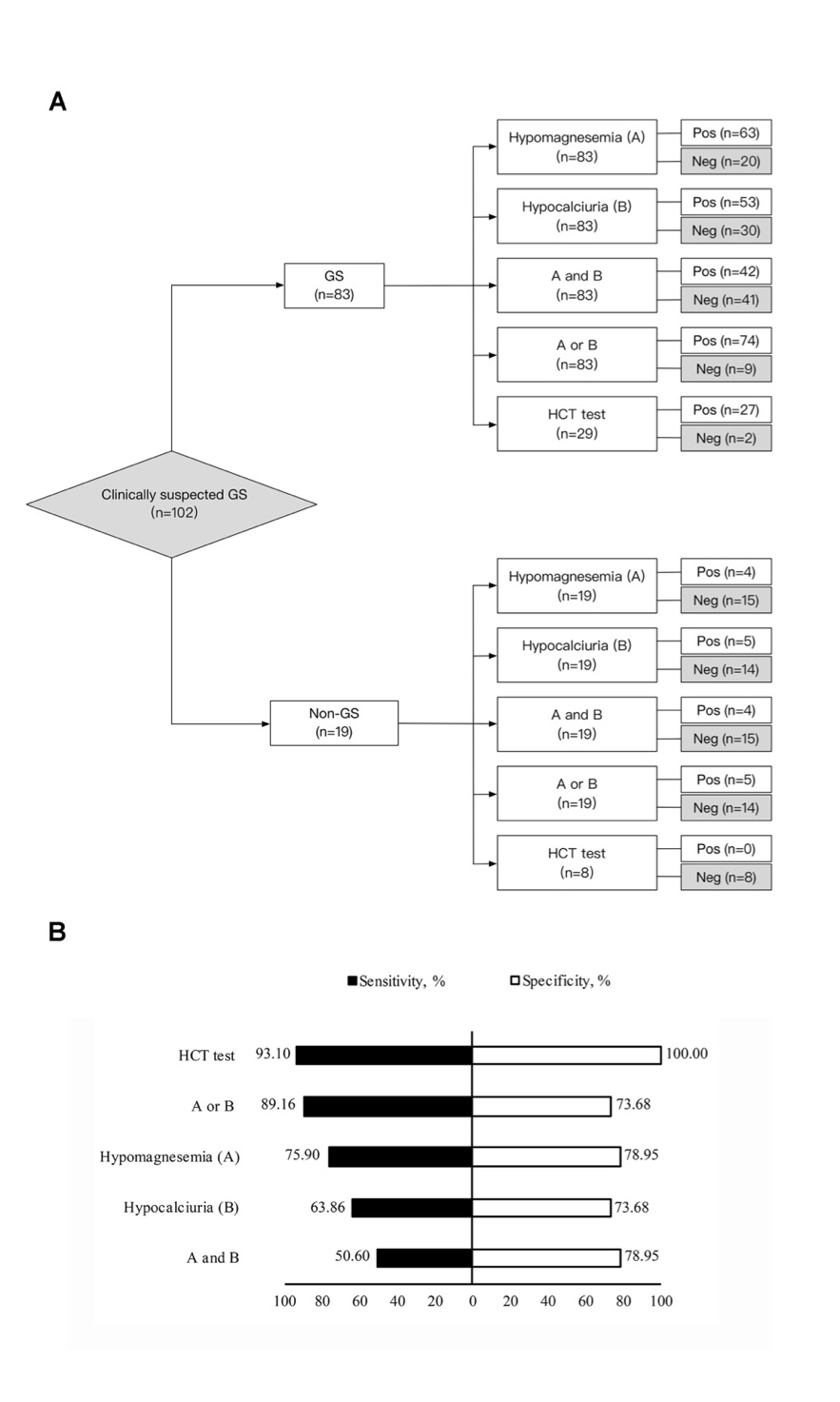


**Supplemental Table 1.** General information and clinical manifestations of genetically confirmed GS patients

| Items | GS with HCT results  (n=29) | GS without HCT results  (n=54) | *P* value |
| --- | --- | --- | --- |
| General information |  |  |  |
| Male | 22 (75.9%) | 26 (48.1%) | 0.015 |
| Age, years | 29.8 ± 15.2 | 31.7 ± 11.7 | 0.511 |
| Onset age, years | 22.3 ± 15.2 | 25.6 ± 12.9 | 0.314 |
| Duration, months | 48.0 (9.5, 120.0) | 40.0 (5.8, 100.5) | 0.670 |
| Symptoms* |  |  |  |
| Muscle weakness | 23 (79.3%) | 36 (67.9%) | 0.273 |
| Fatigue | 18 (62.1%) | 33 (62.3%) | 0.986 |
| Palpitations | 14 (48.3%) | 31 (58.5%) | 0.374 |
| Nocturia | 16 (55.2%) | 19 (35.8%) | 0.091 |
| Paresthesia | 8 (27.6%) | 26 (49.1%) | 0.059 |
| Muscle stiffness/pain | 14 (48.3%) | 18 (34.0%) | 0.204 |
| Carpopedal spasm/tetany | 9 (31.0%) | 18 (34.0%) | 0.787 |
| Thirst | 12 (41.4%) | 13 (24.5%) | 0.113 |
| Polyuria | 6 (20.7%) | 9 (17.0%) | 0.678 |
| Dizziness | 5 (17.2%) | 15 (28.3%) | 0.265 |
| Cramps | 6 (20.7%) | 11 (20.8%) | 0.994 |
| Abdominal pain | 3 (10.3%) | 6 (11.3%) | 1.000 |
| Diarrhea | 3 (10.3%) | 4 (7.5%) | 0.984 |
| Fainting | 2 (6.9%) | 2 (3.8%) | 0.927 |
| Arthralgia | 0 (0.0%) | 4 (7.5%) | 0.327 |
| BMI, kg/m^2^ | 23.11 ± 3.88 | 21.97 ± 4.03 | 0.253 |
| eGFR, ml/min/1.73m^2^ | 119.3 ± 25.3 | 115.0 ± 19.3 | 0.391 |
| SBP, mmHg | 114.2 ± 10.7 | 107.6 ± 12.5 | 0.019 |
| DBP, mmHg | 72.4 ± 10.1 | 71.9 ± 9.9 | 0.827 |

Values are mean ± SD, median (25th, 75th) or n (%). Abbreviations: BMI, body mass index; eGFR, estimated glomerular filtration rate; SBP, systolic blood pressure; DBP, diastolic blood pressure. *n (GS without HCT result) =53, the patient who had suffered drug-induced deafness and was unable to express himself well was excluded from symptom evaluation.

**Supplemental Table 2.** General information and clinical manifestations of genetically confirmed non-GS patients

| Items | Non-GS with HCT result (n=8) | Non-GS without HCT result (n=11) | *P* value |
| --- | --- | --- | --- |
| General information |  |  |  |
| Male | 4 (50.0%) | 5 (45.5%) | 1.000 |
| Age, years | 38.3 ± 12.8 | 27.5 ± 10.4 | 0.059 |
| Onset age, years | 34.6 ± 15.4 | 16.9 ± 13.6 | 0.023 |
| Duration, months | 12.5 (6.5, 51.0) | 60.0 (30.5, 216.0) | 0.112 |
| Symptoms* |  |  |  |
| Muscle weakness | 7 (87.5%) | 5 (55.6%) | 0.294 |
| Fatigue | 6 (75.0%) | 6 (66.7%) | 1.000 |
| Palpitations | 4 (50.0%) | 4 (44.4%) | 1.000 |
| Nocturia | 1 (12.5%) | 4 (44.4%) | 0.294 |
| Paresthesia | 0 (0.0%) | 3 (33.3%) | 0.206 |
| Muscle stiffness/pain | 0 (0.0%) | 2 (22.2%) | 0.471 |
| Carpopedal spasm/tetany | 1 (12.5%) | 5 (55.6%) | 0.131 |
| Thirst | 1 (12.5%) | 2 (22.2%) | 1.000 |
| Polyuria | 0 (0.0%) | 2 (22.2%) | 0.471 |
| Dizziness | 3 (37.5%) | 3 (33.3%) | 1.000 |
| Cramps | 1 (12.5%) | 4 (44.4%) | 0.294 |
| Abdominal pain | 0 (0.0%) | 0 (0.0%) |  |
| Diarrhea | 2 (25.0%) | 2 (22.2%) | 1.000 |
| Fainting | 0 (0.0%) | 1 (11.1%) | 1.000 |
| Arthralgia | 1 (12.5%) | 1 (11.1%) | 1.000 |
| BMI, kg/m^2^ | 22.30 ± 5.13 | 24.28 ± 6.72 | 0.519 |
| eGFR, ml/min/1.73m^2^ | 89.8 ± 19.9 | 109.6 ± 24.1 | 0.075 |
| SBP, mmHg | 115.5 ± 19.1 | 109.8 ± 18.7 | 0.543 |
| DBP, mmHg | 72.5 ± 11.8 | 68.9 ± 10.9 | 0.522 |

Values are mean ± SD, median (25th, 75th) or n (%). Abbreviations: BMI, body mass index; eGFR, estimated glomerular filtration rate; SBP, systolic blood pressure; DBP, diastolic blood pressure. *n (Non-GS without HCT result) =9.

**Supplemental Table 3.** Laboratory biochemical data of genetically confirmed GS patients.

| Items | GS with HCT result  (n=29) | GS without HCT test  (n=54) | *P* value |
| --- | --- | --- | --- |
| minimum serum K*, mmol/L | 2.18 ± 0.33 | 2.22 ± 0.48 | 0.705 |
| minimum serum Mg*, mmol/L | 0.60 ± 0.16 | 0.58 ± 0.15 | 0.589 |
| minimum serum Cl*, mmol/L | 93.0 ± 4.5 | 93.1 ± 4.3 | 0.916 |
| Serum#, mmol/L |  |  |  |
| K | 3.04 ± 0.51 | 3.21 ± 0.47 | 0.126 |
| Na | 137.8 ± 4.4 | 138.1 ± 2.2 | 0.675 |
| Cl | 96.1 ± 4.1 | 96.0 ± 3.4 | 0.904 |
| Mg | 0.67 ± 0.15 | 0.63 ± 0.16 | 0.322 |
| Ca | 2.39 ± 0.17 | 2.42 ± 0.12 | 0.246 |
| P | 1.27 ± 0.24 | 1.25 ± 0.21 | 0.708 |
| 24h Urine, mmol/day | |  |  |
| K | 93.9 (70.3, 118.8) | 96.4 (62.1, 127.6) | 0.924 |
| Na | 225.4 (168.0, 312.3) | 214.5 (165.5, 288.0) | 0.519 |
| Cl | 257.0 (204.1, 307.4) | 238.0 (191.5, 330.0) | 0.558 |
| Mg | 4.68 (3.79, 6.84) | 4.42 (3.76, 5.60) | 0.415 |
| Ca | 1.24 (0.69, 2.16) | 0.82 (0.46, 1.71) | 0.095 |
| P | 19.55 (13.17, 26.08) | 14.24 (10.21, 21.06) | 0.041 |
| Ca/Cr, mmol/mmol | 0.264 (0.098, 0.386) | 0.095 (0.040, 0.226) | 0.006 |
| Arterial blood gas |  |  |  |
| pH | 7.46 ± 0.02 | 7.47 ± 0.03 | 0.071 |
| cHCO_2_^-^, mmol/L | 28.89 ± 3.92 | 29.92 ± 3.79 | 0.246 |
| ABE, mmol/L | 4.48 ± 2.51 | 5.98 ± 3.34 | 0.037 |
| Renin-Angiotensin system | |  |  |
| Renin | 3.10 (1.41, 10.81) | 2.87 (1.43, 12.00) | 0.759 |
| AngII | 294.2 (152.9, 440.7) | 200.3 (142.4, 367.9) | 0.328 |
| Ald | 19.53 (13.98, 24.97) | 19.99 (13.30, 24.80) | 0.822 |
| QTc, ms | 438.5 (418.5, 454.5) | 449.0 (420.3, 476.3) | 0.272 |

Values are mean ± SD, median (25th, 75th) or n (%). *The minimum serum potassium (K), sodium (Na) and chloride (Cl) levels are the minimal levels in the record. #The serum electrolytes levels were measured when the patients visited our hospital for the first time. Abbreviations: ABE, actual base excess; AngII, angiotensin II; Ald, aldosterone; QTc, corrected QT interval.

**Supplemental Table 4.** Laboratory biochemical data of genetically confirmed non-GS patients.

| Items | Non-GS with HCT result (n=8) | Non-GS without HCT result (n=11) | *P* value |
| --- | --- | --- | --- |
| minimum serum K*, mmol/L | 2.43 ± 0.77 | 2.64 ± 0.65 | 0.518 |
| minimum serum Mg*, mmol/L | 0.86 ± 0.20 | 0.79 ± 0.15 | 0.381 |
| minimum serum Cl*, mmol/L | 96.1 ± 6.4 | 94.8 ± 5.9 | 0.668 |
| Serum#, mmol/L |  |  |  |
| K | 3.16 ± 0.51 | 3.33 ± 0.63 | 0.550 |
| Na | 139.9 ± 2.5 | 138.5 ± 2.1 | 0.209 |
| Cl | 99.9 ± 5.1 | 100.1 ± 4.8 | 0.935 |
| Mg | 0.89 ± 0.20 | 0.84 ± 0.18 | 0.611 |
| Ca | 2.33 ± 0.17 | 2.36 ± 0.18 | 0.720 |
| P | 1.22 ± 0.23 | 1.27 ± 0.22 | 0.593 |
| 24h Urine, mmol/day | |  |  |
| K | 58.9 (48.8, 104.6) | 86.3 (25.2, 102.6) | 0.741 |
| Na | 149.0 (88.3, 203.8) | 176.0 (101.0, 248.0) | 0.342 |
| Cl | 168.0 (114.0, 220.0) | 203.1 (133.0, 254.0) | 0.257 |
| Mg | 3.47 (2.48, 6.25) | 4.47 (3.76, 6.94) | 0.327 |
| Ca | 3.28 (2.36, 3.90) | 4.43 (2.28, 5.84) | 0.620 |
| P | 19.23 (12.24, 23.03) | 18.65 (10.49, 24.31) | 1.000 |
| Ca/Cr, mmol/mmol | 0.253 (0.137, 0.668) | 0.410 (0.166, 0.585) | 0.620 |
| Arterial blood gas |  |  |  |
| pH | 7.42 ± 0.03 | 7.43 ± 0.05 | 0.774 |
| cHCO_2_^-^, mmol/L | 26.54 ± 4.39 | 26.85 ± 4.42 | 0.883 |
| ABE, mmol/L | 2.36 ± 3.77 | 2.66 ± 4.35 | 0.881 |
| Renin-Angiotensin system | |  |  |
| Renin | 4.52 (0.91, 10.50) | 0.61 (0.40, 12.00) | 0.315 |
| AngII | 428.6 (80.8, 559.7) | 218.1 (128.5, 800.0) | 0.775 |
| Ald | 18.85 (10.19, 24.54) | 23.90 (20.00, 29.91) | 0.153 |
| QTc, ms | 408.0 (392.8, 425.8) | 404.0 (340.0, 440.5) | 0.927 |

Values are mean ± SD, median (25th, 75th) or n (%). *The minimum serum potassium (K), sodium (Na) and chloride (Cl) levels are the minimal levels in the record. #The serum electrolytes levels were measured when the patients visited our hospital for the first time. Abbreviations: ABE, actual base excess; AngII, angiotensin II; Ald, aldosterone; QTc, corrected QT interval.

**Supplemental Table 5.** Test performances for the diagnosis of Gitelman syndrome by means of the five criteria studied (102 patients).

| Diagnostic performance | Hypomagnesemia (A) | Hypocalciuria (B) | A and B | A or B | HCT test |
| --- | --- | --- | --- | --- | --- |
| Sensitivity, % | 75.90 | 63.86 | 50.60 | 89.16 | 93.10 |
| Specificity, % | 78.95 | 73.68 | 78.95 | 73.68 | 100.00 |
| PV(+), % | 94.03 | 91.38 | 91.30 | 93.67 | 100.00 |
| PV(-), % | 42.86 | 31.82 | 26.79 | 60.87 | 80.00 |
| Kappa value | 0.414 | 0.249 | 0.169 | 0.581 | 0.854 |

Abbreviations: PV(+), positive predictive value; PV(-), negative predictive value; HCT, hydrochlorothiazide.

**Supplemental Table 6.** Diagnosis test performances in 22 new patients with all tests available*.

| Diagnostic performance | Hypomagnesemia (A) | Hypocalciuria (B) | A and B | A or B | HCT test |
| --- | --- | --- | --- | --- | --- |
| Sensitivity, % | 71.43 | 42.86 | 28.57 | 85.71 | 92.86 |
| Specificity, % | 87.50 | 75.00 | 87.50 | 75.00 | 100.00 |
| PV(+), % | 90.91 | 75.00 | 80.00 | 85.71 | 100.00 |
| PV(-), % | 63.64 | 42.86 | 41.18 | 75.00 | 88.89 |
| Kappa value | 0.545 | 0.154 | 0.129 | 0.607 | 0.904 |

*These patients were never reported in our 2015 paper (Jiang L et al. Endocr. Pract. 2015;21(9):1017-25). Among them there are 14 Gitelman syndrome (GS) patients and 8 non-GS patients.

Abbreviations: PV(+), positive predictive value; PV(-), negative predictive value; HCT, hydrochlorothiazide.

**Supplemental Table 7.** Cost analysis for the hydrochlorothiazide test for each patient

| Items | Expenses |
| --- | --- |
| Laboratory analysis* |  |
| 2 serum samples | ¥ 43 × 2 |
| 8 urinary samples | ¥34 × 8 |
| Others |  |
| Hydrochlorothiazide, 50 mg | ¥1 |
| 1.5 L water | ¥4 |
| 1 urinary collector | ¥7 |
| **Total** | ¥370  (about $54 or €47)^#^ |

* Costs of disposables used in sample collection were included.

# US $ and Euro equivalents for CNY at current exchange rate.
